# Metabolic markers GAPDH, PKM2, ATP5B and BEC-index in advanced serous ovarian cancer

**DOI:** 10.1186/1472-6890-13-30

**Published:** 2013-11-19

**Authors:** Elisabet Hjerpe, Suzanne Egyhazi Brage, Joseph Carlson, Marianne Frostvik Stolt, Kjell Schedvins, Hemming Johansson, Maria Shoshan, Elisabeth Åvall-Lundqvist

**Affiliations:** 1 Department of Oncology, Unit for Gynecologic Oncology, Karolinska University Hospital, SE-17176, Stockholm, Sweden; 2 Department of Oncology and Pathology, Cancer Center Karolinska CCK R8:03, Karolinska Institutet, SE-17176, Stockholm, Sweden; 3 Department of Pathology, Karolinska University Hospital, Stockholm, Sweden; 4 Department of Obstetrics and Gynecology, Karolinska University Hospital, Stockholm, Sweden

**Keywords:** Metabolic markers, GAPDH, PKM2, ATP5B, BEC, Ovarian cancer, Immunohistochemistry, Real-time PCR

## Abstract

**Background:**

A deregulated energy metabolism is a hallmark of malignant disease that offers possible future targets for treatment. We investigated the prognostic value of the glycolytic enzymes glyceraldehyde 3-phosphate dehydrogenase (GAPDH) and pyruvate kinase type M2 (PKM2), mitochondrial β-F1-ATPase (ATP5B) and the bioenergetic cellular (BEC) index in advanced ovarian cancer.

**Methods:**

Fresh tumor samples were prospectively collected from 123 patients undergoing primary surgery for suspected advanced ovarian cancer. Of these, 57 met the eligibility criteria; stage IIC-IV, serous or endometrioid subtype, specimens containing ≥ 50% tumor cells and patients receiving platinum-based chemotherapy. An adequate amount of mRNA could be extracted in all but one case, with a resultant study population of 56 patients. Eighty-six percent of cases had serous tumors, and 93% were grade 2–3. GAPDH, PKM2 and ATP5B mRNA- and protein expression was assessed by real-time PCR and immunohistochemistry. We estimated the association with platinum-free interval (PFI) and overall survival (OS) by Cox proportional hazards models. Median follow-up was 60 months.

**Results:**

High GAPDH mRNA levels (HR 2.1, 95% CI 1.0-4.5) and low BEC-index (HR 0.47, 95% CI 0.23-0.95) were both independently associated with shorter PFI. Median PFI for patients with high GAPDH mRNA was 5.0 months compared to 10.1 months for low expression cases (*p* = 0.031). Similarly, median PFI for patients with low BEC-index based on mRNA was 5.3 months compared to 9.8 months for high BEC-index cases (*p* = 0.028).

**Conclusions:**

High GAPDH or low BEC-index mRNA expression indicate early disease progression in advanced serous ovarian cancer.

## Background

Epithelial ovarian cancer (EOC) is a heterogeneous disease comprising several subtypes with very different response to treatment and prognosis. The majority of EOC-patients are detected at advanced stage and the overall 5-year survival rate is 50% or less [1]. Treatment for primary EOC consists of cytoreductive surgery and platinum-taxane combination chemotherapy. Despite emerging knowledge of subtype-specific variations in tumor biology and chemo-responsiveness, EOC is still treated as one entity. The high-grade serous carcinomas account for 60–70% of diagnosed tumors. This subtype is, although often initially platinum-sensitive, associated with a poor prognosis. Identification of EOC subtype-specific prognostic markers is important for development of new targeted therapies.

A reprogrammed energy metabolism has recently been designated a “new” hallmark of cancer [2]. These metabolic changes, including increased aerobic glycolysis, decreased oxidative phosphorylation and increased glutaminolysis and fatty acid-metabolism, are known features of cancer, which often correlate with an overall poor prognosis [3,4]. Several glycolytic enzymes, for example glyceraldehyde 3-phosphate dehydrogenase (GAPDH) and pyruvate kinase M2 (PKM2), have been reported overexpressed in cancer [5-8]. First introduced by Cuezva and co-workers in 2002 [3], a bioenergetic cellular index (BEC-index), based on the protein levels of mitochondrial β-F1-ATPase (ATP5B, the catalytic subunit of the rate-limiting enzyme of oxidative phosphorylation), heat-shock protein 60 (HSP60, a mitochondrial chaperone), and glycolytic GAPDH, has been shown to be of prognostic value in several cancer types such as colon, lung and breast carcinomas [3,9,10]. The expression of above metabolic markers has not yet been studied in EOC.

We have previously shown, in cell lines and ovarian cancer cells from ascites, that inhibitors of glycolysis can potentiate the effects of different chemotherapeutics, including platinum compounds [11,12]. In the present study we investigated the mRNA and protein expression of GAPDH, PKM2 and ATP5B in 56 advanced serous or endometrioid ovarian cancers, to test their ability to predict length of platinum-free interval and survival. We also assessed, at the mRNA-level, the prognostic significance of the BEC-index.

## Methods

### Patients

We prospectively collected fresh tumor specimens from patients undergoing surgery for epithelial ovarian, fallopian tube or primary peritoneal cancer. Patients were operated at Karolinska University Hospital, Stockholm, Sweden, between April 2003 and July 2008. They all gave informed consent and the Ethics Committee at Karolinska Institutet, Stockholm, approved the study.

**Figure 1:**
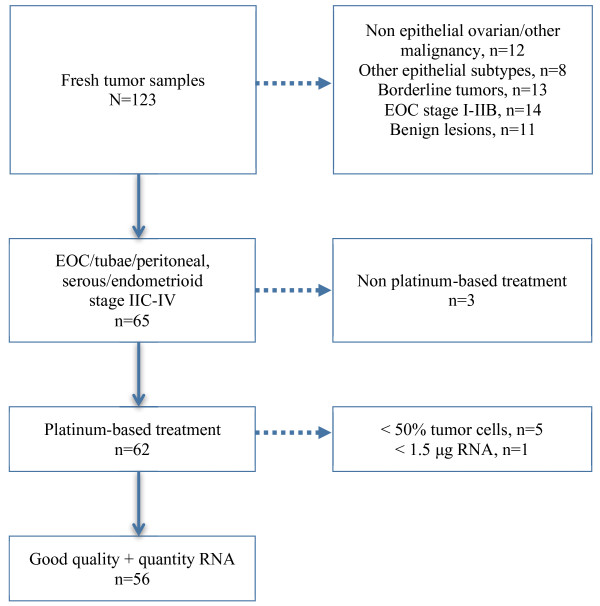
Flow Chart.

Tumor and, if present, ascites samples were obtained from 123 patients of which 62 fulfilled the study’s inclusion criteria, i.e. age ≥ 18 years, FIGO stage IIC-IV serous or endometrioid ovarian, fallopian tube or primary peritoneal cancer (Figure 1). Also, patients had to be scheduled for platinum-based chemotherapy. Tumors were classified and graded according to WHO standards [13]. Patient baseline characteristics, treatment and relapse data were prospectively collected in Case Report Forms (CRFs). All but four patients underwent primary debulking surgery aiming at cytoreduction to microscopic disease. In the remaining cases only biopsy sampling was done. At the time of study, the staging procedure for ovarian cancer did not include lymphadenectomy.

### Tumor sample collection, processing and RNA extraction

The collection and processing of fresh tumor biopsies has been previously described in detail [14]. For solid tumor specimens, imprints were prepared for pathological review. Only samples with ≥ 50% tumor cells were included in the study, and five of 62 cases were thus excluded. We did smear preparations to assess tumor cell yield from ascites, with the same cut-off of 50% tumor cells as for biopsies. Ascites samples were processed using the Lymphoprep™ (Axis-Shield, Oslo, Norway) density gradient separation method [15] for enrichment of tumor cells. We used the RNeasy Midi Kit™ (Qiagen, Hilden, Germany) for RNA extraction. RNA quality was assessed using Bioanalyzer 2100 (Agilent Technologies, Santa Clara, CA). An adequate quantity (≥1.5 μg) and quality (RIN 8–10) RNA could be extracted in all but one case. Thus, the present study population consists of 56 patients (Figure 1). In five cases the RNA yield from the ascites sample was better than that from the corresponding solid tumor, and therefore used in the analysis.

### Real-time PCR

RNA was reverse transcribed by Superscript III (Invitrogene AB, Stockholm, Sweden). We used the ABI 7500 computerized system (Applied Biosystems, Foster City, CA) and SYBR Green™ for amplification reactions. Relative quantity expression values were calculated by the ΔΔCt method using GenEx software (MultiD, Gothenburg, Sweden). Primer sequences are listed in Additional file 1. HPRT1 (hypoxanthine guanine phosphoribosyltransferase 1) and Β2-microglobulin were used as reference genes. Universal Human Reference RNA (Stratagene, Santa Clara, CA) served as positive control. Due to technical failure in one case, PCR analyses were performed in 55 of 56 tumor samples.

For analyses of GAPDH, PKM2 and ATP5B real-time PCR results, we divided cases into three equally sized groups according to their relative quantity of mRNA expression, and the group with lowest expression (one third of patients, n = 18) was then compared to cases with higher expression (two thirds of patients, n = 37). A cut-off at one third was chosen because it approximates the proportion of patients still alive at median follow-up. The BEC-index was calculated, at the mRNA-level, by dividing the ATP5B:HSP60 ratio with the GAPDH expression value. For this, we used recently published real-time PCR data for HSP60 mRNA [14]. The third of cases with the highest BEC-index value (n = 19) was compared to cases with lower values (n = 36).

### Immunohistochemistry

Formalin-fixed, paraffin-embedded tumor blocks could be obtained in 54 of 56 cases. Immunohistochemistry was performed as previously described [14]. In brief, following heat-induced antigen retrieval, primary antibodies (Additional file 2) were incubated at 8° overnight. Reaction products were visualized using the Vectastain Elite ABC kit (Vector Laboratories, Burlingame, CA). Negative controls were produced by omission of the primary antibody. A breast carcinoma specimen served as positive control for GAPDH and PKM2, and for ATP5B a colon carcinoma sample was used.

Three observers (EH, SE and JC), blinded for clinical data, independently evaluated all slides by assessing the whole tumor area. The maximum staining intensity of tumor cells was scored 0-3+, and the percentage of cancer cells thus stained estimated. Cases were dichotomized into low and high expression groups. Cut-offs were set arbitrarily to make groups equally sized; for GAPDH: low expression, 1+ or < 50% of tumor cells staining 2+, and high expression, 3+ or ≥ 50% staining 2+, for PKM2: low, 1-2+ or < 20% of tumor cells staining 3+, and high, ≥ 20% staining 3+, and for ATP5B: low, staining intensity of tumor cells 1-2+, and high, staining intensity 3+ (regardless of proportion). Discrepancies between observers were found for 13% of examined GAPDH-slides, 6% of PKM2-slides and 15% of ATP5B-slides, in which cases consensus was reached on further review.

### Statistics

Statistical analyses were performed using STATA 11.2 software. The platinum-free interval (PFI) was defined as the time interval from last course of primary platinum-based chemotherapy (end of treatment, EOT) to documented disease progression, death, or last follow-up. Overall survival (OS) was defined as the time from diagnosis to death or last follow-up. We estimated PFI and OS by the Kaplan-Meier method [16] and curves were compared using Wald’s test. Cox proportional hazards regression models [17], adjusted for standard confounding risk factors age, stage, grade and postoperative residual tumor, were used for multivariate analyses. P-values below 0.05 were considered statistically significant.

## Results

### Patients

Patients were monitored until 31 March 2011 with a median follow-up of 59.9 (30.2-82.8) months. Clinical and pathological characteristics are presented in Table 1. Over 85% of patients had poorly differentiated (grade 3) carcinomas, and 89% had FIGO stage IIIC-IV disease. In 70% of cases the postoperative tumor residuals measured at least 10 mm. All patients received platinum-based chemotherapy. Of 49 evaluable patients (with measurable disease at start of chemotherapy), 82% responded to treatment. Relapse within 6 months from EOT was detected in 25 of 56 cases (45%). Median PFI was 7.2 months, and median OS 34.6 months. At study closure, 19 patients (34%) were alive, with no evidence of disease in 9 cases (16%).

**Table 1:** **Clinical characteristics of 54 patients**^**a **^**in relation to immunohistochemical staining for GAPDH, PKM2 and ATP5B**

	**GAPDH**		**PKM2**		**ATP5B**	
	***n *** **= 54**		***n *** **= 54**		***n *** **= 54**	
**Characteristic**	low <50% 2+	high ≥50% 2+	low <20% 3+	high ≥20% 3+	low 1-2+	high 3+
	*n* = 26	*n* = 28	*n* = 26	*n* = 28	*n* = 26	*n* = 28
	N (%)	N (%)	N (%)	N (%)	N (%)	N (%)
**Age at diagnosis (years)**						
**Median 64.5 (39–83)**	NA	NA	NA	NA	NA	NA
**Diagnosis**						
**Epithelial ovarian (*n *** **= 41)**	22 (54)	19 (46)	23 (56)	18 (44)	21 (51)	20 (49)
**Fallopian tube (*n *** **= 10)**	4 (40)	6 (60)	4 (40)	6 (60)	4 (40)	6 (60)
**Peritoneal (*n *** **= 3)**	0	3 (100)	0	3 (100)	1 (33)	2 (67)
**FIGO stage**						
**IIC (*n *** **= 2) - IIIB (*n *** **= 3)**	2 (40)	3 (60)	1 (20)	4 (80)	5 (100)	0
**IIIC (*n *** **= 42)**	21 (50)	21 (50)	21 (50)	21 (50)	18 (43)	24 (57)
**IV (*n *** **= 7)**	3 (43)	4 (57)	4 (57)	3 (43)	3 (43)	4 (57)
**Subtype**						
**Serous (*n *** **= 47)**	22 (47)	25 (53)	19 (40)	28 (60)	23 (49)	24 (51)
**Endometrioid (*n *** **= 7)**	4 (57)	3 (43)	7 (100)	0	3 (43)	4 (57)
**Grade of differentiation**^**b**^						
**High (*n *** **= 4)**	2 (50)	2 (50)	1 (25)	3 (75)	2 (50)	2 (50)
**Moderate (*n *** **= 4)**	2 (50)	2 (50)	2 (50)	2 (50)	2 (50)	2 (50)
**Poor (*n *** **= 46)**	22 (48)	24 (52)	23 (50)	23 (50)	22 (48)	24 (52)
**Postop residual tumor size**						
**0 mm (*n *** **= 6)**	3 (50)	3 (50)	0	6 (100)	4 (67)	2 (33)
**1-10 mm (*n *** **= 10)**	4 (40)	6 (60)	3 (30)	7 (70)	5 (50)	5 (50)
**>10 mm (*n *** **= 38)**	19 (50)	19 (50)	23 (61)	15 (39)	17 (45)	21 (55)
**1**^**st **^**line Chemotherapy**						
**Carboplatin + paclitaxel (*n *** **= 47)**	NA	NA	NA	NA	NA	NA
**Other platinum based (*n *** **= 7)**						
**Time from EOT to recurrence/progression**						
**< 6 months (*n *** **= 25)**	10 (40)	15 (60)	13 (52)	12 (48)	8 (32)	17 (68)
**≥ 6 months (*n *** **= 28)**	16 (57)	12 (43)	13 (46)	15 (54)	17 (61)	11 (39)
**Unknown (*n *** **= 1)**	0	1 (100)	0	1 (100)	1 (100)	0
**Survival**						
**Alive, no evidence of disease (*n *** **= 8)**	4 (50)	4 (50)	2 (25)	6 (75)	6 (75)	2 (25)
**Alive, with disease (*n *** **= 9)**	6 (67)	3 (33)	4 (44)	5 (56)	5 (56)	4 (44)
**Death from disease (*n *** **= 36)**	16 (44)	20 (56)	19 (53)	17 (47)	14 (39)	22 (61)
**Death from other cause (*n *** **= 1)**	0	1 (100)	1 (100)	0	1 (100)	0

*Abbreviations*: FIGO, Federation Internationale de Gynecologie et d’Obstetrique, EOT, End of treatment, NA, Not applicable.

^a^Tumor blocks could be retrieved in 54 of 56 cases.

^b^Grade of differentiation according to WHO international histological classification of tumors.

### GAPDH

**Figure 2:**
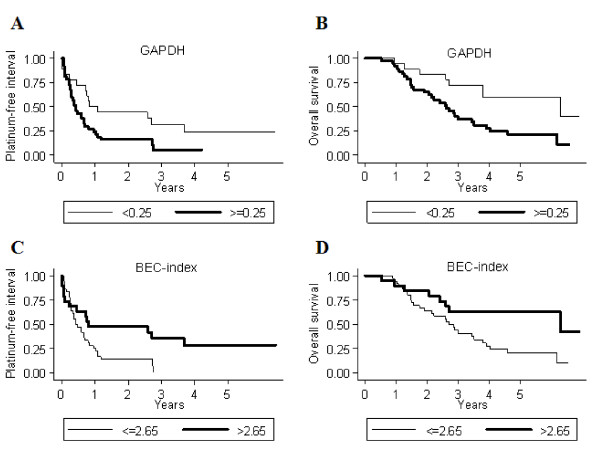
**Platinum-free interval (PFI) and overall survival (OS) according to GAPDH and BEC-index mRNA expression.** Kaplan-Meier curves for GAPDH **(A and B)** and BEC-index based on mRNA **(C and D)**. Patients with high GAPDH had shorter PFI (*p* = 0.031) and OS (*p* = 0.015), whereas patients with high BEC-index had longer PFI (*p* = 0.028) and OS (*p* = 0.033).

Eighteen patients had a tumor GAPDH mRNA expression of less than 0.25, and 37 cases expressed at least 0.25 relative to reference genes and positive control. Kaplan-Meier curves for PFI and OS, based on GAPDH mRNA expression, are shown in Figure 2A and B. The median PFI was significantly shorter in the group with high expression compared to the low expression group (5.0 and 10.1 months, respectively, *p* = 0.031). Table 2 shows uni- and multivariate analyses of PFI and OS. In univariate analysis, high GAPDH mRNA expression was associated with shorter PFI (HR 2.1, 95% CI 1.1-4.0, *p* = 0.031) and OS (HR 2.8, 95% CI 1.2-6.5, *p* = 0.015). In multivariate analysis, high GAPDH mRNA remained significant for shorter PFI (HR 2.1, 95% CI 1.0-4.5, *p* = 0.043) but not for OS (HR 2.3, 95% CI 1.0-5.6, *p* = 0.063).

**Table 2:** Uni- and multivariate analyses

		**Expression**	**Univariate analysis**		**Multivariate analysis**^**a**^	
			**HR (95% CI)**	***p***	**HR (95% CI)**	***p***
GAPDH	PFI	low (<0.25), *n* = 18	1		1	
		high (≥0.25), *n* = 37	2.1 (1.1-4.0)	0.031	2.1 (1.0-4.5)	0.043
	OS	low (<0.25), *n* = 18	1		1	
		high (≥0.25), *n* = 37	2.8 (1.2-6.5)	0.015	2.3 (1.0-5.6)	0.063
PKM2	PFI	low (<0.70), *n* = 18	1		1	
		high (≥0.70), *n* = 37	1.2 (0.7-2.3)	0.55	1.5 (0.8-2.8)	0.27
	OS	low (<0.70), *n* = 18	1		1	
		high (≥0.70), *n* = 37	1.1 (0.5-2.2)	0.87	1.2 (0.6-2.6)	0.60
ATP5B	PFI	low (<0.31), *n* = 18	1		1	
		high (≥0.31), *n* = 37	1.7 (0.9-3.3)	0.089	1.8 (0.9-3.6)	0.097
	OS	low (<0.31), *n* = 18	1		1	
		high (≥0.31), *n* = 37	2.6 (1.1-5.9)	0.025	2.3 (1.0-5.3)	0.062
BEC-index	PFI	low (≤2.65), *n* = 36	1		1	
		high (>2.65), *n* = 19	0.46 (0.23-0.92)	0.028	0.47 (0.23-0.95)	0.035
	OS	low (≤2.65), *n* = 36	1		1	
		high (>2.65), *n* = 19	0.42 (0.20-0.94)	0.033	0.49 (0.22-1.31)	0.088

*Abbreviations*: HR, hazard ratio, CI, confidence interval.

^a^Multivariate model with adjustment for age, FIGO stage, grade and postoperative residual tumor size.

Platinum-free interval (PFI) and overall survival (OS) in relation to mRNA expression of GAPDH, PKM2, ATP5B and BEC-index.

**Figure 3:**
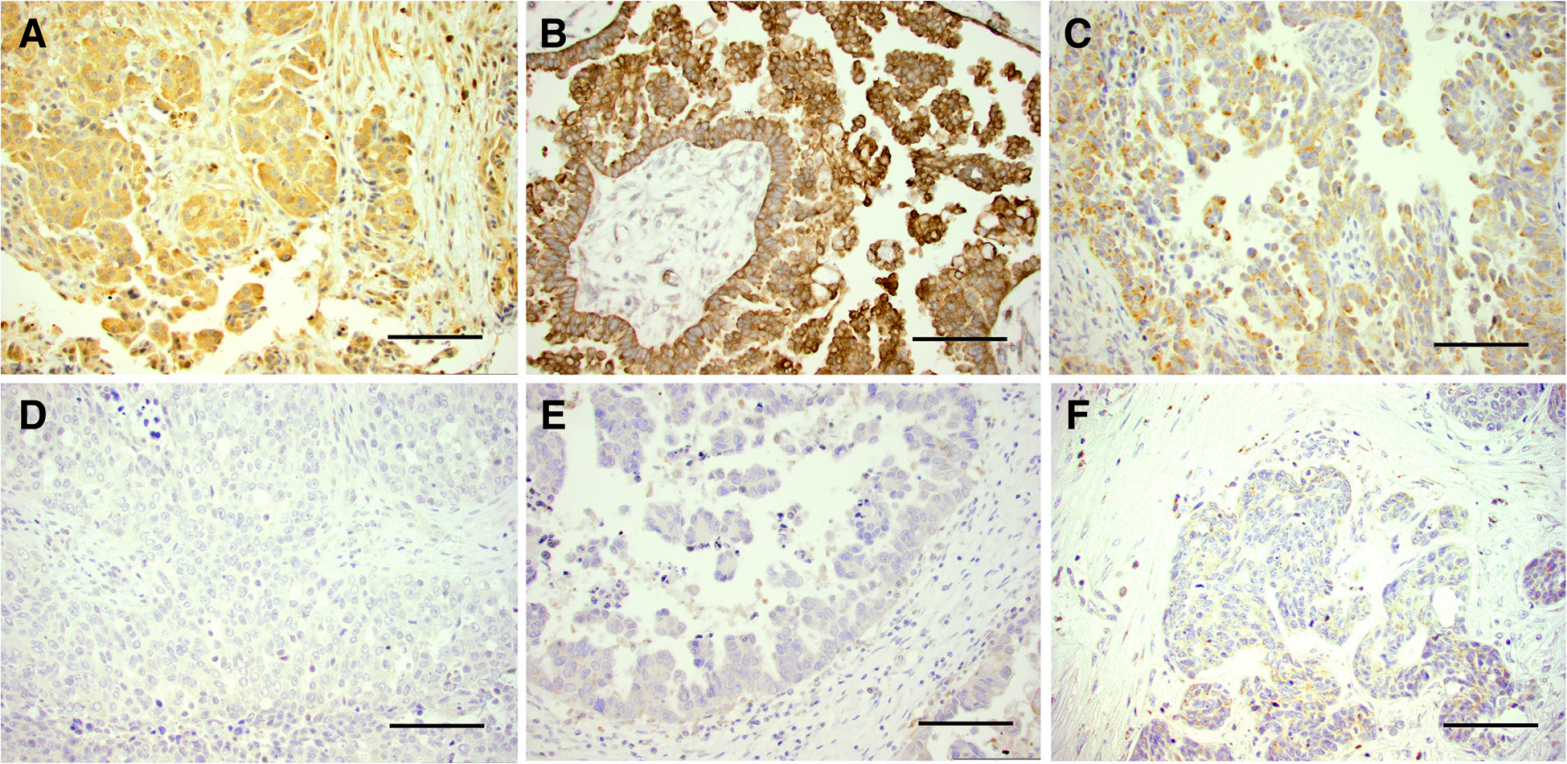
**Immunohistochemical staining for GAPDH, PKM2 and ATP5B. A** and **D** shows examples of high and low GAPDH-reactivity, **B** and **E** shows high and low PKM2-reactivity and **C** and **F** exemplifies high and low ATP5B-reactivity. Bar = 100 μm.

GAPDH protein expression by IHC was low in 26 and high in 28 of 54 cases (Table 1, Figure 3). GAPDH-reactivity was predominantly cytoplasmatic, but in 22 cases (41%) we also found nuclear localization of the enzyme. There were no statistically significant differences in PFI or survival between groups with high- or low GAPDH protein expression (data not shown).

### PKM2

Eighteen patients had a tumor PKM2 mRNA expression of less than 0.70, and 37 cases had higher mRNA expression. There were no statistically significant differences in PFI or survival between groups with high or low mRNA expression (Table 2).

Twenty-six cases had low PKM2 protein expression and in 28 cases the expression was high (Table 1, Figure 3). The assessed PKM2-reactivity was cytoplasmatic, but in 11 cases (20%) nuclear localization of the protein was observed in mitotic cells. The immunoreaction does not provide any information whether the enzyme present is in its dimeric or more glycolytically active tetrameric form. There were no statistically significant differences in PFI or survival between high and low PKM2 protein expressing groups.

### ATP5B

Eighteen patients had a tumor ATP5B mRNA expression of less than 0.31, and in 37 cases the mRNA expression was higher. Uni- and multivariate analyses of PFI and OS are shown in Table 2. In univariate analysis, high ATP5B mRNA expression predicted poor OS (HR 2.6, 95% CI 1.1-5.9, *p* = 0.025), but it did not remain significant in multivariate analysis (HR 2.3, 95% CI 1.0-5.3, *p* = 0.062).

Twenty-six cases had low ATP5B protein expression and in 28 cases the expression was high (Table 1, Figure 3). ATP5B-reactivity was cytoplasmatic and often granular. In univariate analysis, a high ATP5B protein expression predicted short PFI (HR 1.9, 95% CI 1.0-3.4, *p* = 0.039), but it did not reach significance in the multivariate model (data not shown).

### BEC-index

Thirty-six patients had an mRNA-based tumor BEC-index of ≤ 2.65 and 19 cases had higher expression. Kaplan-Meier curves for PFI and OS, based on BEC-index mRNA expression, are shown in Figure 2C and D. The median PFI was significantly longer in the group with high compared to the group with low BEC-index (9.8 and 5.3 months, respectively, *p* = 0.028). Table 2 shows uni- and multivariate analyses for PFI and OS. Multivariate analysis showed a high BEC-index mRNA to independently predict longer PFI (HR 0.47, 95% CI 0.23-0.95, *p* = 0.035). Also, univariate analysis indicated high BEC-index to predict longer survival (HR 0.42, 95% CI 0.20-0.94, *p* = 0.033), but it did not remain significant in multivariate calculations.

## Discussion

Our data indicate that high GAPDH as well as low BEC-index based on mRNA are associated with early disease progression in patients with advanced high-grade serous adenocarcinomas of the ovary, fallopian tube or peritoneum. To our knowledge, no other study has investigated the prognostic impact of these markers in ovarian cancer.

GAPDH, a tetramer of four identical 37-kDa subunits, is a key glycolytic enzyme present in all tissues. It has commonly been used as a house-keeping reference gene/protein for expression analyses. However, this practice has recently been challenged due to findings of GAPDH playing a role in cancer pathogenesis. In a recent review GAPDH expression was reported deregulated in e.g. cancers of the lung, breast, colon, pancreas, liver, kidney and prostate [18].

In agreement with our results, Révillion and co-workers reported reduced OS and relapse-free survival among breast cancer patients with enhanced GAPDH mRNA expression as assessed by real-time PCR [19]. The study included 404 unselected breast cancer patients with a median follow-up of 82 months. In multivariate analyses, GAPDH was not an independent prognostic factor, but its expression was inversely correlated to oestradiol and progesterone receptor concentrations, young age and grading. Thus, GAPDH mRNA expression in breast cancer seems to reflect tumor aggressiveness. In the present study, GAPDH mRNA expression was an independent marker for early tumor progression in a cohort of ovarian cancer patients with poor prognosis. Also, our results demonstrate that GAPDH should not be used as reference RNA in ovarian cancer.

In contrast to a recently published study in breast cancer we found no association between GAPDH protein expression and platinum-free interval or OS. Descotes et al. used 2D-electrophoresis and Western blotting in 41 node-negative breast cancers of which 20 had metastatic relapse and 21 had no metastatic recurrence [6]. The former group had significantly higher GAPDH expression. Both our studies are based on small cohorts and should be considered as exploratory. In addition to tumor type, the methodological approaches also differed. There are several possible biological explanations to the apparent discrepancy between our GAPDH mRNA and protein data, e. g. posttranslational regulation. A plausible reason can also be the limited sensitivity of immunohistochemistry, which requires major differences in expression for visual detection.

Taking both glycolytic and oxidative capacity into account, a low BEC-index has been shown to predict shorter survival in colon, lung and breast carcinomas [3,9,10]. Although we used mRNA instead of protein expression data, the results of the present study in ovarian cancer are in accordance with these reports. Apart from GAPDH and ATP5B, the BEC-index is also influenced by the expression of the mitochondrial multifunctional chaperone HSP60. We have earlier shown HSP60 overexpression to predict poor outcome in serous ovarian cancer [14], and the BEC-index data from the present study probably reflects both the tumor material’s GAPDH and HSP60 expression.

ATP5B-expression *per se* has also been investigated, but the results seem contradictory. At the protein level, high ATP5B has been shown to predict longer survival in colon cancer [3,20]. In contrast, it was found overexpressed [21] and associated with poor survival [10] in breast cancer. In the present study, high ATP5B mRNA expression in ovarian cancer was associated with worse OS, but was not found to have independent prognostic importance in multivariate analysis (*p* = 0.062).

PKM2 has been linked to poor survival in signet ring cell cancer and to advanced stage in colorectal cancer [7,8]. However, the results from our work indicate that PKM2 might not provide prognostic information in ovarian cancer.

The mechanisms by which the deregulated metabolic enzymes in cancer mediate the observed poor prognosis are complex. Rapidly proliferating cells have a high demand for energy and glycolytic intermediates for anabolic processes. Some glycolytic enzymes also directly participate in pathways of proliferation and apoptosis [22-24]. In addition, PKM2 has been implicated in platinum-resistance in ovarian cancer cell lines [25], and down-regulation of ATP5B protein expression has *in vitro* been shown to induce 5-FU-resistance in colon cancer [26]. However, targeting breast cancer cell lines with ATP5B-inhibitor Aurovertin B decreases proliferation [21].

We present a homogeneous clinical material of advanced, mainly high-grade serous cancers, in which all patients had peritoneal spread. In this uniform cohort with dismal prognosis, GAPDH and BEC-index mRNA could distinguish groups with different time to relapse. In line with this, other studies have supported the notion that there are different subpopulations of high-grade serous carcinomas [14,27].

This study is limited by its small size, so our results cannot be generalized to all patients with advanced serous carcinomas. Its strengths are the prospective study design and application of CRFs for collection of data. We also used fresh tumor samples to minimize analytical errors and our statistical analyses were adjusted for known risk factors.

## Conclusions

Our findings indicate that GAPDH and BEC-index may identify groups of advanced high-grade serous carcinomas with different prognosis. However, before transferring these results into clinical practice, validation in large independent cohorts is needed. Targeting metabolic enzymes could be a future strategy to improve survival in this group of patients with poor prognosis.

## Competing interests

The authors declare that they have no competing interests.

## Authors’ contributions

EH conceived the study, participated in the PCR and IHC analyses, analyzed data and wrote the manuscript. SE helped design the study and analyzed data. Pathologist JC evaluated IHC. MFS performed the PCR. KS performed surgery and provided tumor samples. HJ did statistical analyses. MS helped in study design and provided conceptual input. EÅL designed the study, analyzed data and revised the manuscript. All authors read and approved the final manuscript.

## Pre-publication history

The pre-publication history for this paper can be accessed here:

http://www.biomedcentral.com/1472-6890/13/30/prepub

## Supplementary Material

Additional file 1 Primers for real-time PCR. Click here for file

Additional file 2 Primary antibodies for immunohistochemistry. Click here for file
