# Supplementary material for: Metabolic markers GAPDH, PKM2, ATP5B and BEC-index in advanced serous ovarian cancer
Source: BMC Clin Pathol. 2013 Nov 19;13:30. doi: 10.1186/1472-6890-13-30 (PMC3874631; doi:10.1186/1472-6890-13-30)
Supplement: Additional file 1 — Primers for real-time PCR. [file 1472-6890-13-30-S1.docx]

**Additional file 1. Primers for real-time PCR**.

| GAPDH | forward  reverse | 5’-GAAGGTGAAGGTCGGAGT-3’  5’-GAAGATGGTGATGGGATTTC-3’ |
| --- | --- | --- |
| PKM2 | forward  reverse | 5’-ATGAGTACCATGCGGAGACC-3’  5’-TGTCTAGAGCCACAGCAACG-3’ |
| ATP5B | forward  reverse | 5’-TCACCCAGGCTGGTTCAGA-3’  5’-AGTGGCCAGGGTAGGCTGAT-3’ |
| HSP60 | forward  reverse | 5’-CACCGTAAGCCTTTGGTCAT-3’  5’-CTTGACTGCCACAACCTGAA-3’ |
| HPRT (reference gene) | forward  reverse | 5’-GACACTGGCAAAACAATGCAGAC-3’  5’-GGTCCTTTTCACCAGCAAGCT-3’ |
| B2M (reference gene) | Commercially available human B2M primers (TAATA Biocenter, Gothenburg, Sweden) | |
