# Supplementary material for: Metabolic markers GAPDH, PKM2, ATP5B and BEC-index in advanced serous ovarian cancer
Source: BMC Clin Pathol. 2013 Nov 19;13:30. doi: 10.1186/1472-6890-13-30 (PMC3874631; doi:10.1186/1472-6890-13-30)
Supplement: Additional file 2 — Primary antibodies for immunohistochemistry. [file 1472-6890-13-30-S2.docx]

**Additional file 2. Primary antibodies for immunohistochemistry**.

| Epitope | Source | Catalogue no | Dilution |
| --- | --- | --- | --- |
| GAPDH | Santa Cruz Biotechnology, CA | V-18 SC-20357 | 1:200 |
| PKM2 | Cell Signaling, Darmstadt, DE | 3198 | 1:400 |
| ATP5B | Atlas Antibodies, Stockholm, SE | HPA1520 | 1:3000 |
